# The Role of Collagen XVII in Cancer: Squamous Cell Carcinoma and Beyond

**DOI:** 10.3389/fonc.2020.00352

**Published:** 2020-03-19

**Authors:** Virginia A. Jones, Payal M. Patel, Frederick T. Gibson, Adriana Cordova, Kyle T. Amber

**Affiliations:** Skin Immunology Laboratory, Department of Dermatology, University of Illinois at Chicago, Chicago, IL, United States

**Keywords:** collagen XVII, literature review, skin cancer, cancer, ectodomain shedding

## Abstract

Alterations in the extracellular matrix (ECM) likely facilitate the first steps of cancer cell metastasis and supports tumor progression. Recent data has demonstrated that alterations in collagen XVII (BP180), a transmembrane protein and structural component of the ECM, can have profound effects on cancer invasiveness. Collagen XVII is a homotrimer of three α1 (XVII) chains. Its intracellular domain contains binding sites for plectin, integrin β4, and BP230, while the extracellular domain facilitates interactions between the cell and the ECM. Collagen XVII and its shed ectodomain have been implicated in cell motility and adhesion and are believed to promote tumor development and invasion. A strong association of collagen XVII ectodomain shedding and tumor invasiveness occurs in squamous cell carcinoma (SCC). Aberrant expression of collagen XVII has been reported in many epithelial cancers, ranging from squamous cell carcinoma to colon, pancreatic, mammary, and ovarian carcinoma. Thus, in this review, we focus on collagen XVII's role in neoplasia and tumorigenesis. Lastly, we discuss the importance of targeting collagen XVII and its ectodomain shedding as a novel strategy to curb tumor growth and reduce metastatic potential.

## Introduction

Cancer is a leading cause of death in developed countries (1). In the United States, cancer ranks as the second leading cause of overall mortality, with non-melanoma skin cancer (squamous cell carcinoma and basal cell carcinoma) as the most common form (2). Between the years 2007–2011, an estimated 4.9 million adults were treated for skin cancer, an increase of 1.5 million cases from 4 years prior (3). While incidence cannot be precisely determined, the data suggests that skin cancer incidence is likely increasing (2). Extensive research efforts are continuously underway to identify new targets for cancer treatment. Tumor microenvironment is of particular interest, particularly for invasiveness (4–6). The mechanism of tumor progression from the perspective of tumor stroma has provided a new outlook on cancer development. Following this model, studies have shown that extracellular matrix (ECM), historically considered a structural barrier to tumor cell migration, likely facilitates the first steps of cancer cell metastasis and supports tumor progression (6, 7).

Collagens within the tissue stroma represent a valuable target in cancer biology. At present, 28 triple helical collagen proteins with a variety of structures and functional properties have been discovered in humans (8). Here we will discuss collagen XVII (BP180), a type II hemidesmosomal transmembrane protein and a structural component of the dermoepidermal junction (9, 10). Given notable recent discoveries in COL17A1 expression in cancers, as well as the role of aberrant collagen XVII ectodomain shedding in squamous cell carcinoma, it appears that collagen XVII may play a particularly important role in epithelial cancer growth and invasiveness.

Cell biological analyses propose that collagen XVII functions as a cell-matrix adhesion molecule by stabilizing the hemidesmosome complex. Stabilization occurs by the projection of collagen XVII beneath hemidesmosomes in epithelia, particularly basal keratinocytes, thereby, mediating anchorage to the underlying basement membranes (10–13). Beyond its structural role, collagen XVII is presumed to play a role in skin inflammation, cell migration, and differentiation in physiologic and pathologic states (9, 14).

## Structure of Collagen XVII, Interactions of its Subunits, and Function in Membrane Adhesion

**Figure 1:**
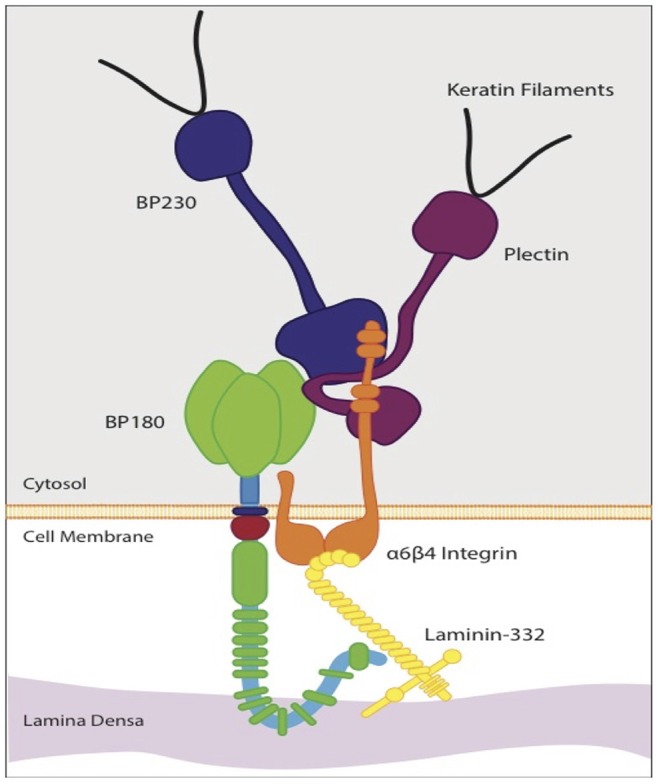
Schematic diagram of the molecular organization of a hemidesmosorne at the dermoepidermal basement membrane. The intracellular plaque is associated with transmembrane adhesion molecules, including integrins and transmembrane type XVII collagen (BP 180). It contains plectin and BP 230 (BPAG1). Type XVII collagen regulates expression and function of laminin- 332. The interaction between laminin-332 and the extracellular portions of a6β4 integrin stabilizes hemidesmosomes. This interaction is essential for hemidesmosome formation and epithelial adhesion.

Collagen XVII is a 180-kD type II transmembrane protein and structural component of the dermoepidermal anchoring complex, which facilitates adhesion of keratinocytes and certain other epithelial cells to the underlying basement membrane (10). Its gene, COL17A1, has been mapped to chromosome 10q24.3 (15, 16). Collagen XVII along with integrin α6β4, CD151, plectin, and BP230 are components of type I hemidesmosomes (HDs), which are responsible for cell-stromal adherence, cell polarization, and spatial organization of tissue architecture (10, 16). Collagen XVII and integrin α6β4 are transmembrane proteins that bind laminin-332 in the basement membrane (17). Collagen XVII acts as an anchor protein connecting extracellular and intracellular hemidesmosomal proteins (18). The importance of collagen XVII for the integrity of the dermoepidermal junction is demonstrated in inherited skin diseases caused by mutations in the COL17A1 gene, including junctional epidermolysis bullosa (JEB), and in numerous subepidermal blistering skin disorders with autoantibodies to collagen XVII (BP180), such as bullous pemphigoid (BP), linear IgA bullous dermatosis, lichen planus pemphigoides, and pemphigoid gestationis [Figure 1; (19, 20)].

Collagen XVII is a homotrimer of three α1 (XVII) chains, each with a globular cytosolic amino (N-) terminal domain, a short transmembrane stretch, and a flexible-rod extracellular carboxy (C-) terminal domain (10). The intracellular domain (ICD) of collagen XVII consists of 466 amino acids (aa) while the transmembrane domain (TD) and extracellular domain (ECD) are 23 and 1,008aa, respectively (21). The ICD lies in the outer plaque of the HD. The ECD extends into the lamina densa, an electron-dense zone, and loops back into the lamina lucida, an electron clear zone (22).

**Figure 2:**
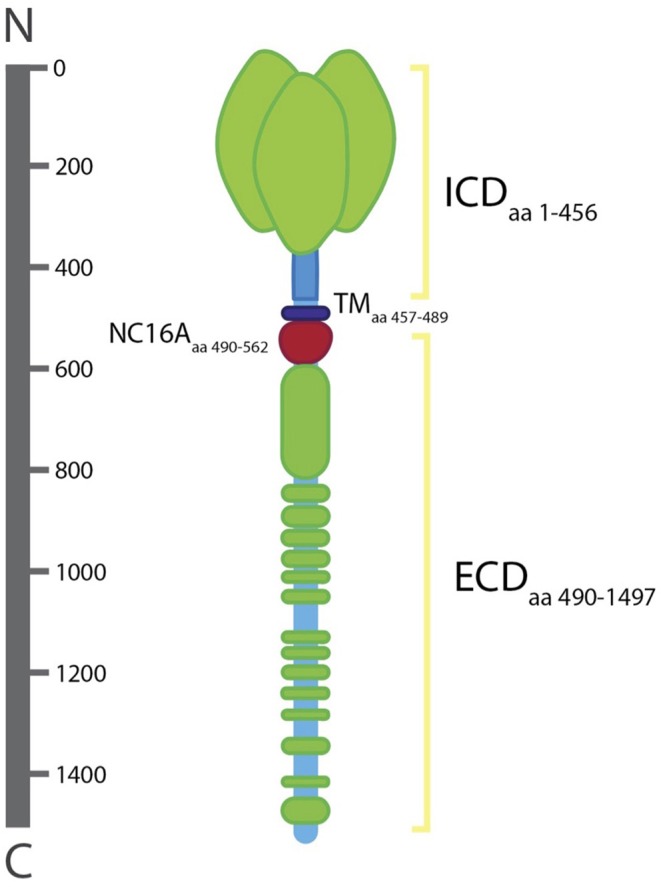
Structure of collagen XVII. The intracellular domain (lCD; aa 1–456), transmembrane domain (TD; aa 457–489), and extracellular domain (ECD; aa 490–1497). The ECD includ es the NC16A domain (aa 490–562). The ICD lies in the outer plaque of the HD and the ECD spans the lamina Iucida, extends to the lamina densa, and loops back into the lamina Iucida.

The ICD contains binding sites for plectin, integrin β4, and BP230 (16). The localization of collagen XVII into HDs has been shown to be dependent on its interaction with integrin β4, which has at least two binding sites in the ICD including one in the first 230aa stretch and another present in aa 231–401 [Figure 2; (23, 24)]. In the absence of integrin β4, HDs fail to form (24). Collagen XVII's ICD is also responsible for the recruitment and binding of BP230 and plectin, both of which are suggested to bind via a region of 280 aa residues (aa 180–460) (24). The recruitment and binding of BP230 and plectin mediate the linkage of HDs with the keratin cytoskeleton of epithelial cells, composed of keratin 5 and 14, and are necessary for the correct formation of HDs (24, 25).

Collagen XVII's ECD facilitates interactions between the cell and the ECM by providing a link between the cytoplasmic structural components and the ECM (26). The ECD of 15 collagenous subdomains characterized by Gly-X-Y repeat sequences (COL1-COL15) separated by 16 non-collagenous subdomains (NC1-NC16) (10, 16). The ECD is constitutively shed from the cell surface leading to the formation of ~120-kD protein fragment, known as the ectodomain, which is integrated into the basement membrane. The ectodomain is essential for proper basement membrane formation and is suggested to play a role in cell motility, adhesion, and differentiation (27, 28).

### Ectodomain Shedding

Under physiologic conditions, ectodomain shedding is catalyzed by the ADAMs (a disintegrin and metalloproteinase) sheddase family (28, 29). ADAMs are transmembrane proteins that promote ectodomain shedding of collagen XVII into the extracellular space by cleaving between the plasma membrane and the COL15 subdomain within the NC16A linker domain (aa 490–562), which contains sheddase recognition and cleavage sites (30).

The NC16A subdomain has an α-helical coiled-coil structure and has been identified as the likely site for association and initiation of triple helix formation in collagen XVII (31). Contrary to fibrillar collagen where coiled coils are most often found in the C-terminal propeptides and function to initiate trimerization and triple helix formation from the C to N terminus, collagen XVII, along with other membrane-associated collagens, contain coiled coils in the N-terminal non-collagenous domains, and thus, undergo triple helix formation from the N to C terminus (30, 32). The physiological cleavage sites are eight to eleven aa C-terminal from the coiled-coils within the region Leu^524^-Gly^526^ (30, 33).

ADAM9 and 10 have been identified as the major sheddases involved in ectodomain shedding (29). While ADAM17 was originally thought to be the major sheddase involved, it was shown that ADAM17 may play a more indirect role, as direct stimulation of ADAM17 activity did not lead to ectodomain shedding (29). The influence of sequence specificity, physical length of the substrate from proteolysis site, and substrate secondary structure on sheddase function and ectodomain shedding remains an area of controversy (34, 35). Ectodomain shedding can be increased by mutations in the coiled-coil motifs Val^492^-Ile^505^, which induces non-physiologic shedding C-terminally from the furin motif, a non-physiologic cleavage site (30). Likewise, deletion of the Ala^528^-Glu^547^, an area outside of the physiologic cleavage site, prevents ectodomain shedding (36). As such, structural motifs appear to play an important role in conformation-dependent susceptibility to sheddase related ectodomain cleavage.

The shed ectodomain interacts dynamically with the ECM including binding partners integrin α6 and laminin-332 (26). Integrin α6 and laminin-332 are both necessary for the localization of collagen XVII and stabilization of HDs (26, 37). Integrin α6 is proposed to bind to the juxtamembranous NC16 subdomain, and laminin-332 is proposed to bind near the extracellular carboxyl-terminal domain (37). The binding of autoantibodies with the ectodomain (ex. NC16A) can lead to the development of bullous pemphigoid, resulting in skin blistering and dermatitiss (20, 38, 39).

## Functional Roles of Collagen XVII

Beyond its structural role in the hemidesmosome complex, collagen XVII is presumed to play a role in cell migration and differentiation in disease states (9). This postulation comes from studies that examine overexpression and knock-out models of collagen XVII in different cell lines and disease states.

### The Role of Collagen XVII in Cell Differentiation

Ablation of COL17A1 has been proven to result in the loss of self-renewal and differentiation capacity of hair follicle stem cells (HFSCs) (13, 40). It has been demonstrated that COL17A1 is required for the maintenance of quiescence and immaturity, which thereby, maintains the self-renewing capabilities of HFSCs (13). In COL17A1- null mice, forced expression of COL17A1 in basal keratinocytes and HFSCs rescues melanocyte stem cells from premature differentiation while restoring TGF-β signaling (13). Additional studies have found that stem cell aging of HFSCs begins with DNA damage recruitment of neutrophil elastase and subsequent COL17A1 proteolysis, demonstrating that COL17A1 in HSFCs orchestrates stem-cell aging (40). Lastly, COL17A1 has been shown to play a major role in differentiation of hair-follicle associated pluripotent stem-cell markers (HAP)—which are nestin-expressing HFSC (41), into epidermal keratinocytes. This expression of COL17A1 is increased in HAP stem-cell markers only during periods of differentiation (42). Taken all together, these studies demonstrate the role of collagen XVII in cell differentiation in HFSC. Its role in regulating differentiation in other lines remains to be determined.

### The Role of Collagen XVII in Cell Migration

Collagen XVII is also postulated to play a role in cell migration. Collagen XVII-deficient keratinocytes display increased spreading on laminin-332, increased expression of the integrin β4 subunit, and activated PI3K and Rac1, resulting in the formation of multiple unstable leading edges (lamellipodia) (17). These findings suggest collagen XVII is involved keratinocyte adhesion and directed motility coordination by interfering with integrin dependent PI3K activation (17, 43).

Furthermore, release of the ectodomain of collagen XVII from the cell surface has been associated with decreased keratinocyte motility, while addition of the ectodomain of collagen XVII inhibits motility in migrating cells (27, 44). It is postulated that an optimum adhesive strength is required for cell migration, with overly weak adhesions resulting in insufficient cell traction, while overly strong adhesions inhibit cell locomotion (44, 45). All together, these results demonstrate that collagen XVII inhibits keratinocyte migration, while collagen XVII's shed ectodomain leads to stabilization and cell immobilization (44).

This theory is proven in patients with junctional epidermolysis bullosa (JEB), a disorder characterized by mutations in the COL17A1 gene resulting in the absence of collagen XVII or expression of a structurally altered protein leading to subepidermal blistering and immature HDs (44, 46–48). The lack of a strong adhesion in these collagen XVII deficient keratinocytes is reflected by increased motility compared to controls, and is in line with the hypothesis of weakening basal keratinocytes adherence to the BM (44, 46, 47). Lastly, collagen XVII may coordinate keratinocyte migration during wound healing. Collagen XVII-deficient cells have proven to be hypermobile with faster healing of scratch wounds than controls (44). Conversely, wound closure is slower with the addition of exogenous ectodomain of collagen XVII (27).

### The Role of Collagen XVII in Skin Inflammation

It is well-known that dysfunction of collagen XVII, either through genetic or autoantibody insult, leads to subepidermal blistering and skin inflammation. Nonetheless, a direct role of collagen XVII in skin inflammation is unclear. In a humanized NC16A mouse, mutation induced dysfunction of NC16A in basal keratinocytes (termed ΔNC16A), leads to development of spontaneous skin inflammatory disease (49). These mice exhibit severe pruritus, dysfunctional skin barrier, infiltrating immune cells, increased serum IgE levels, and high expression of thymic stromal lymphopoietin (TSLP) (49). Severe pruritus is not mediated by adaptive immunity or histamine, rather, it is dependent on increased expression of TSLP (49).

## Involvement of Collagen XVII in Cancer Development

The malignant transformation of cancer is defined by its ability to invade normal tissues. As an essential component of the ECM, collagen XVII plays a significant role in forming a barrier against cell infiltration. Beyond its function as a cell-matrix adhesion molecule, collagen XVII's role in cancer biology has only recently been a point of investigation. The altered expression and regulation of collagen XVII, along with its effects on signaling cascades indicate its role in tumor development and invasion. Understanding this protein's role in tumor progression may be promising target in tumorigenesis.

### Collagen XVII as It Pertains to Skin Cancer

#### Squamous Cell Carcinoma

In normal epidermis, collagen XVII expression ceases as keratinocytes migrate upwards and differentiate. SCC, however, demonstrates collagen XVII overexpression, suggesting de-differentiation (50, 51). Immunohistochemical analysis exhibited stronger immunoreaction of collagen XVII ectodomain in the primary SCC tumor and associated metastases (51). This is similarly noted in atypical keratinocytes of actinic keratoses and SCC *in situ* (51, 52). Viral mediated RNA interference knockdown of collagen XVII and/or β4 disrupted the migration and invasion of less aggressive SCC cells (52). Of note, no impact was observed on HSC-3, a highly invasive cell with upregulated collagen XVII (52).

Collagen XVII positivity has also been widely noted in head and neck SCC, especially within those originating from the oral cavity and larynx (53, 54). In a sample of tongue SCC samples, collagen XVII and laminin-332 were found to be co-localized at the invasive tumor fronts protruding into the surrounding tissue, and proteolytic shedding of collagen XVII enhanced its functioning as a chemotactic agent through its effects on transmigration of HSC-3 cells (50).

At the transcriptional level, overexpression of collagen XVII mRNA was found in the invasive tumors via reverse-transcriptase polymerase chain reaction (RT-PCR) at the epidermal level and within epithelial tissues. RT-PCR and northern hybridization confirmed the enhanced expression of collagen XVII in SCC (7, 55). Among oral keratinocytes stimulated with the tumor promoting phorbol ester (56), a 1.5-fold induction in collagen XVII mRNA expression was noted (53). In addition, quantitative PCR also confirmed COL17A1 expression and upregulation by transfection of miR-203a-3p inhibitor in oral SCC lines (57). The miRNA used in the analysis, miR-103a-3p, was found to bind human 3′UTRs of *COL17A1* (58).

There is also a strong association with collagen XVII ectodomain shedding and tumor invasiveness in SCC (59). To investigate this role, a non-sheddable collagen XVII mutant with a deletion in the linker domain spanning over cleavage sites (60) was retrovirally introduced in SCC-25 cells, a cell used to assess aggregate size in SCC. Prevention of collagen XVII shedding resulted in smaller aggregate sizes, colony formation, and decreased Matrigel invasion, while collagen XVII re-expression restored tumorigenicity (59).

#### Basal Cell Carcinoma

Non-radioactive *in situ* hybridization revealed the presence of collagen XVII mRNA among basal keratinocytes of solid basal cell carcinoma (BCC) and the tumor islands of superficial BCC, while it was absent in the basal cells of healthy epidermis (9). Overall, immunostaining revealed decreased collagen XVII expression among the peripheral cells in solid and keratotic BCCs and the basal keratinocytes of invasive tumor fronts in superficial BCCs (9). A later study was unable to replicate this finding as collagen XVII immunostaining was mostly negative in the basal cell cancer islets, except in some dispersed spindle cells (51). Moreover, further evaluation is needed to confirm the role of collagen XVII in BCC progression.

#### Neural Crest Tumors

Collagen XVII expression was noted in the cells of neural crest origin and proliferating tissue melanocytes, but not in benign melanocytic tumors (55). All subtypes of melanoma were strongly positive whereas the nevus cells in nevoid melanoma were negative for significant staining (55). Furthermore, immunostaining of collagen XVII was statistically correlated with the invasive phenotype and the vertical “Breslow” thickness of melanomas (55). *In vitro* treatment of melanoma cells with aa 507–529 sequence specific antibody against collagen XVII endodomain promoted apoptosis, reduction of tumor cell proliferation, and cell adhesion (55). Sequencing of COL17A1 gene from melanoma cDNA detected several point mutations and in-frame deletions in the ectodomain coding region, suggesting the contribution of post-translational degradation in ectodomain deficiency (55).

*In vivo* models using skin specific-NC16A dysfunctional mice with B16 melanoma cells support the regulatory role of keratinocyte collagen XVII on melanoma tumorigenesis (61, 62). When compared to the controls, the experimental models showed increased levels of tumor volume and lymphatic metastasis (62). Collagen XVII dysfunction results in immediate inflammation, previously identified as a hallmark for cancer, through the influx of chemokines which further recruit myeloid derived suppressor cells (MDSCs) (61, 63). Decreasing the levels of MDSCs through treatment with specific antibodies resulted in the reduction of tumor volume, and the rate of metastatic development, demonstrating the role collagen XVII plays in curbing tumor invasion via regulation of MDSC infiltration (62). As some studies have noted an association between bullous pemphigoid and melanoma, particularly after immunotherapy, this remains a potentially relevant interaction (64, 65).

### Collagen XVII as It Pertains to Other Cancers

In addition to squamous cell carcinoma and other skin cancers, dysregulation of collagen XVII appears to occur in numerous other cancers as outlined in Table 1.

**Table 1:** Collagen XVII as it pertains to cancer development.

**Cancer**	**Protein**	**mRNA**	**References**
Squamous cell carcinoma	+	+	(51–53)
Actinic keratosis	+		(51, 52)
Melanoma	+		(55)
Basal cell carcinoma	+/–	+	(9)
Pancreatic carcinoma	*		(14)
Thyroid cancer	+		(66)
Colorectal cancer	↑	↑	(67, 68)
Lung cancer	↑	↑	(69, 70)
Nasopharyngeal cancer		↓	(71)
Salivary gland cancer	–	–	(72)
Cervical cancer	↑		(7, 73)
Breast cancer	↓		(7, 74)

+, expression; –, absence; ↑, upregulation; ↓, downregulation; *The 120 kD shed ectodomain is the predominant protein form detected.

#### Pancreatic Cancer

Immunohistochemical visualization has demonstrated that mature type hemidesmosome 1 (collagen XVII + integrin α6β4; HD-1) can be found in the human pancreatic ductal epithelium, with subsequent disassembly during pancreatic carcinogenesis (14). The HD-1 breakdown is part of the phosphoinositide 3-kinase dependent induction of matrix-metalloprotease 9 (MMP-9), which cleaves collagen XVII and enables integrin α6β4 to promote cell migration and invasion (14, 52).

#### Colorectal Cancer

The role of collagen XVII as in colorectal cancer progression has been studied extensively. Overexpression of full-length collagen XVII and elevated mRNA levels have been found in samples of colorectal carcinoma (67, 68). Collagen XVII overexpression was associated with higher TNM staging and correlated with an infiltrative growth pattern, tumor budding, metastasis, and decreased survival rates (67). *In vitro*, the overexpression of murine collagen XVII promoted the invasiveness of colon cancer cells through the Matrigel (67). COL17A1 gene expression was upregulated via the PP2A-S727STAT3 mediated pathway (68). Inhibition of this pathway in colorectal cancer metastasis blocked suspension survival, sphere formation, tumor initiation, and metastasis, reinforcing the role of collagen XVII as an important prognostic factor in colorectal cancer patients (68).

#### Lung Cancer

In a sampling of non-small cell lung cancers, elevated expression of collagen XVII was seen in the stromal environment and was associated with increased metastatic potential (69). Microarray analysis revealed the upregulation of collagen XVII and elevated mRNA levels in spheroid cultures demonstrating its importance in the maintenance of the mesenchymal transition and metastatic ability in lung cancer like stem cells (70). Collagen XVII's shed ectodomain appears to stabilize laminin-332, in turn leading to activation of FAK/AKT/GSK3β. Ultimately, this leads to suppression of snail ubiquitination-degradation, propagating the epidermal-mesenchymal transition (70). Blockade of the signaling pathway also decreased the metastatic potential of lung metastases *in vivo* (70). Similar findings were demonstrated in A549 lung adenocarcinoma cell lines in adherent culture with induced collagen XVII overexpression (75). In these cells, the collagen XVII-laminin-332 pathway induced the FAK/AKT/GSK3β/β-catenin pathway leading to Oct4-HK2 activation and induction of lung cancer stem cells (CSCs)-like features (75). These include sphere formation, pluripotency marker expression, tumorigenic potential, metastasis, and metabolic reprogramming of CSC which is essential for CSC survival and maintenance (75). Notably, increased collagen XVII, Oct4, and HK2 are associated with poorer prognosis (75). A separate study examining lung tumorigenesis analyzed mice deficient for both Mob1A and Mob1B in bronchioalveolar epithelial cells as lung adenocarcinomas arise from bronchioalveolar stem cells (BASCs) (76). Mob1A/1B deficient adult mouse showed decreased mRNA levels of the COL17A1 gene in their lung epithelial cells resulting in subsequent suppression of tumor initiation (76).

#### Salivary Gland Cancer

Undifferentiated carcinoma (UDC) of the salivary gland, an uncommon disease responsible for <2% of major salivary gland tumors, carries histopathologic features similar to those noted in poorly differentiated nasopharyngeal carcinoma (NPC) (72, 77, 78). AMC-HN-9 cell lines are derived from UDC of the parotid gland and are arranged into sheets of atypical epithelial cells with an underlying fibrous stroma (72, 79). Collagen XVII is not expressed in AMC-HN-9 cells, and cytogenetics has not demonstrated gross deletions or rearrangements affecting collagen XVII gene loci (72). Previous studies have demonstrated that collagen XVII is expressed in normal salivary tissue epithelium (80). Therefore, the exact mechanism under which collagen XVII expression is lost in AMC-HN-9 cells remains uncertain (72).

#### Cervical Cancer

Aberrant expression of BP180 was first identified in the cervix immunohistochemically using a validated murine monoclonal antibody exhibiting specific reactivity to skin hemidesmosomes (73). A latter study confirmed collagen XVII overexpression in cervical tumors through immunohistochemistry, along with increased local dissemination and metastasis (7). Moreover, COL17A1 promoter was found to be hypomethylated in cervical carcinoma, head and neck squamous cell carcinoma, lung squamous cell carcinoma, and lung adenocarcinoma resulting in subsequent collagen XVII overexpression (7, 51, 55, 81, 82).

#### Breast Cancer

Immunohistochemistry on ductal breast cancers detected that collagen XVII is underexpressed (74). This underexpression is correlated with higher TNM staging, increased invasion, and postmenopausal status (7). The opposed direction of expression in breast cancer may be due to hypermethylation of the COL17A1 promoter (7). These findings suggest that the COL17A1 promoter methylation status dictates the direction of collagen XVII expression, with reduced expression leading to poorer prognosis (7).

#### Nasopharyngeal Cancer

Reduced mRNA expression of collagen XVII is observed in nasopharyngeal carcinoma cells (NPC) examined by RT-PCR (72). Previous studies have shown that downregulation of HD components (such as collagen XVII) can facilitate migration of carcinoma cells through detachment from the basement membrane (71, 83), suggesting that downregulation of HD protein expression may play a pivotal role in neoplastic progression to early invasion (84, 85). Downregulation of collagen XVII in NPC cell lines suggests that it is a shared feature of the malignant transformation of NPC (71).

## Future Perspectives: Collagen XVII and Cancer Treatment

Collagen XVII assumes various roles in epithelial biology including but not limited to cell differentiation, cell migration, and skin inflammation. In a variety of tumors, genetic aberrations in COL17A1, full-length collagen XVII overexpression, or the accumulation of its proteolytic components have been linked to increased invasiveness of tumors (52, 62, 67, 69). For example, ectodomain shedding of COL XVII is catalyzed by ADAM9 and ADAM10 (27, 36, 86), and ectodomain release promotes tumor invasion (59). Selective blockage of collagen XVII ectodomain shedding was achieved in *in vitro* treatment of SCC-25 cells with the monoclonal NC16A-1 antibody raised against (aa 490–523) (87), and also demonstrated reduced tumor growth (59). Likewise, HSC-3 cell cultures treated with NC16A-1 antibody exhibited reduced invasion, confirming the role of shedding in SCC metastasis (59). In light of these findings, identifying therapeutic inhibitors of collagen XVII ectodomain shedding serves as a promising therapeutic avenue in SCC. While this *in vivo* and *in vitro* approach offers a potentially targeted means of inhibiting ectodomain shedding, several clinical studies have assessed protease inhibition, which presumably impacted ectodomain shedding.

MMP-9 is involved in the proteolytic cleavage of collagen XVII and is implicated in various forms of carcinomas including pancreatic, invasive cutaneous SCC, and mucosal SCC (14, 59, 88, 89). While MMP9 represents a tempting pharmacologic target for cancer therapy due to its role in facilitating extracellular matrix (ECM) degradation (90, 91), past RCTs from the 1990's to the early 2000's deemed MMP-9 inhibitors (MMPI) such as marimastat, unsuccessful in reducing tumor burden or mortality, and found increased rates of adverse effects (92–94). Previous generation MMPIs were flawed in their target to the MMP catalytic site, which is highly conserved throughout the MMP family (94), and resulted in drugs that blocked MMP-mediated proteolysis but could not selectively inhibit the specific pathologically overexpressed MMPs (94).

However, novel highly selective MMPIs offer the potential for more targeted therapy, with presumably fewer off target-related adverse events (95). Of these, Andecaliximab/GS-5745, an MMP-9 inhibitor prevents zymogen activation by binding MMP-9 functionally between the propeptide and catalytic domains (96), and also serves as an allosteric inhibitor (97). Andecaliximab has shown promise in phase I clinical trials of gastric adenocarcinoma (98). However, a phase II study failed to support the addition of andecaliximab to PD-1 inhibitor therapy (99), and a phase III study failed to support the addition of andecaliximab to the mFOLFOX6 regimen (100).

Given the importance of collagen XVII overexpression and ectodomain shedding in the tumorigenesis of SCC and other cancers, it serves as a promising treatment target. While more refined MMPIs may improve on previously ineffective treatments, multiple proteases may act on the same substrate, and there may additionally be compensation by untargeted metalloproteases. As such, targeted therapy directly blocking the substrate such as with NC16A-1 antibody may represent an innovative treatment. The absence of significant phenotype in mice unable to shed their collagen XVII ectodomain, suggests that inhibition of shedding may alternatively be a more tolerated therapeutic target (60).

## Author Contributions

KA: conceptualization. VJ, PP, FG, AC, and KA: writing-original draft preparation. VJ and FG: figures. PP: table. VJ, PP, FG, and KA: writing-review and editing.

### Conflict of Interest

The authors declare that the research was conducted in the absence of any commercial or financial relationships that could be construed as a potential conflict of interest.
